# Mental Affections: An Introduction to the Study of Insanity

**Published:** 1900-06

**Authors:** 


**Mental Affections: an Introduction to the Study of Insanity.** By JOHN MACPHERSON, M.D. Pp. x., 380. London: Macmillan and Co., Limited. 1899.

The author does not lay claim to the production of a text-book on insanity, but merely to a somewhat expanded edition of lectures delivered to his class. We think that, even as the book stands, it is more fitted for the advanced student of mental disease than for the needs of the ordinary curriculum, and we should welcome certain enlargements of its scope which would equip it for use as a text-book. With this premise, we cordially commend the volume before us; it gives ample evidence of conscientious preparation, careful and independent thought, and comprehensive literary research. There are not, nor should there be in a work more especially designed for students, frequent attempts to expound original views, but the author for the most part treats his subject with a freshness and freedom which engross the attention. The histological and morbid anatomy sections are, we think wisely, dealt with rather briefly; the more so as no plates illustrate the letterpress; within the limited space a careful epitome of recent views has been given. The chapter on Cortical Localisation appears to be mainly an abstract of Fleschig's researches; we note that the gyrus fornicatus and subiculum cornu Ammonis are quoted as portions of the limbic lobe, but it is nearly certain from Elliott Smith's work that the true limbic lobe does not include either of these parts. At page 112 the statement is found of an ocular sensation being conveyed *viâ* the optic nerves to the cortical centre in the first temporal convolution; this is doubtless an unwitting error, the said region being auditory in function.

In considering the pathogenesis of disordered mental action, the author propounds his working hypothesis, which deals with the increased or diminished resistance of the neuron and dissociation of grouped neurons, normally in equilibrium. The functional abeyance of a neuron involves the lessened activity of the nerve-elements in physiological continuity with it, and this condition is assumed to underlie melancholia, there being underaction and increased resistance in higher ideational centres and inhibition of lower sensory and motor centres. These states are correlated to the mental pain, slow reaction, and diminished sensibility. It is not clear to us how agitated forms of melancholia are explicable upon this hypothesis, but the author later on discusses this question; he considers that the intellectual centres, though resistive, are by no means wholly inactive, the pent-up activity finds vent through other channels, and if the malady be acute is expressed in motor agitation. In mania, though a more profound abeyance in consciousness is asserted, there is supposed to be hyperactivity and lessened resistance in superior ideational planes, and consequent removal of inhibition from lower centres "which pour their stimuli unrestrainedly into the ideation centres." The term inhibition, we presume, must in the first case be understood as want of stimulus; in the second, in the usually accepted sense of control. The author, then, apparently regards the deeper reductions of mania as connected with over-excitation of high ideational centres, not as a more profound abeyance in their activities. An alteration in the dynamic state of ideational centre-neurons, *plus* or *minus*, is supposed to account for alternating conditions of excitement or depression.

The section on Cerebral Dissociation is well and attractively written, and obsession, delusion, and hallucination are defined, analysed, and illustrated with care.

The clinical section of the book is thoroughly good, and the student's grasp of the essential types of insanity is not made uncertain by a voluminous and entirely needless classification. Paranoia is written of with a masterly knowledge of its characteristic features, and in a simple yet graphic way. The author places general paralysis amongst the toxic disorders; he believes an important part in its causation to be played by syphilis, but only as a para-syphilitic poison. The tissue changes may result from continued action of this toxin, or from premature degeneration due to previous toxicity of blood, and, further, from products (cholin) of nerve tissue disintegration. The volume is produced with an extreme care most creditable to author and publishers.

